# Microbial Fabrication of Zinc Oxide Nanoparticles and Evaluation of Their Antimicrobial and Photocatalytic Properties

**DOI:** 10.3389/fchem.2020.00778

**Published:** 2020-09-30

**Authors:** Devendra Jain, Shivani, Ali Asger Bhojiya, Himmat Singh, Hemant Kumar Daima, Mandeep Singh, Santosh Ranjan Mohanty, Bjorn John Stephen, Abhijeet Singh

**Affiliations:** ^1^Department of Molecular Biology and Biotechnology, Maharana Pratap University of Agriculture and Technology, Udaipur, India; ^2^Department of Agriculture and Veterinary Sciences, Mewar University, Chittorgarh, India; ^3^Material Research Centre, Malviya National Institute of Technology, Jaipur, India; ^4^Amity Center for Nanobiotechnology and Nanomedicine (ACNN), Amity Institute of Biotechnology, Amity University Rajasthan, Rajasthan, India; ^5^All India Network Project on Soil Biodiversity-Biofertilizers, ICAR-Indian Institute of Soil Science, Bhopal, India; ^6^School of Science, RMIT University, Melbourne, VIC, Australia; ^7^Department of Biosciences, Manipal University Jaipur, Jaipur, India

**Keywords:** zinc oxide nanoparticles, zinc tolerant bacteria, antimicrobial, photocatalytic dye degradation, physiochemical

## Abstract

Zinc oxide (ZnO) nanoparticles have attracted significant interest in a number of applications ranging from electronics to biomedical sciences due to their large exaction binding energy (60 meV) and wide bandgap of 3.37 eV. In the present study, we report the low-cost bacterium based “eco-friendly” efficient synthesis of ZnO nanoparticles by using the zinc-tolerant bacteria *Serratia nematodiphila*. The physicochemical characterization of ZnO nanoparticles was performed by employing UV-vis spectroscopy, XRD, TEM, DLS, Zeta potential, and Raman spectroscopy. The antimicrobial and antifungal studies were investigated at different concentrations using the agar well-diffusion method, whereby the microbial growth rate decreases with the increase in nanoparticle concentration. Further, photocatalytic performance studies were conducted by taking methyl orange (MO) as a reference dye.

## Introduction

The field of nanotechnology has revolutionized almost all the aspects of human life, and their potential applications in industries such as agriculture, medicine, cosmetics, electronics, and textiles have recently gained momentum. Due to nanoparticles large surface to volume ratio, their surface reactivity is greatly enhanced resulting in extraordinary antimicrobial, antifungal, catalytic, and wound healing properties which are generally not observed in their bulk counterpart (Siddiqi et al., 2018; Marimuthu et al., 2020). Generally, nanomaterials can be synthesized either through physical, chemical, or green synthesis methods. The physical methods, viz., inert gas condensation, physical vapor deposition, laser and flame spray pyrolysis, electro spraying techniques, and melt mixing are costly and require high throughput equipment, while the chemical methods, viz., sol-gel synthesis, micro emulsion technique, hydrothermal synthesis, polyol synthesis, and plasma enriched vapor deposition utilizes chemicals that produce toxic by-products that could be harmful to the body or the ecosystem. However, the green synthesis route offers an eco-friendly, cost-effective, and time-efficient mode of nanomaterial synthesis, which is being intensively investigated as an alternative to the conventional mode of synthesis.

Various types of nanomaterials are known to have specific properties that can be exploited in several industries. Metallic nanoparticles such as zinc oxide nanoparticles are currently being explored due to their properties such as large binding energy and high piezoelectric properties. Further, they are deemed to be biocompatible and non-toxic increasing its industrial potential. From a biological perspective, ZnO is considered an important metal oxide which can be involved in a range of metabolic processes like the synthesis of carbohydrates, lipids, nucleic acid and protein, and their degradation (Jain et al., 2013; Vidyashree, 2016). Zinc deficiencies lead to several of abnormalities in plants such as smaller leaves, chlorosis, stunted growth, and spikelet sterility (Hafeez et al., 2013). It also plays an active role in producing an important growth hormone, auxin, that enhances output and quality in several plants such as wheat and rice (Hu et al., 2003; Cakmak, 2008). Due to the large surface to volume ratio, ZnO nanoparticles facilitate better interaction with microbes thus possess an excellent antifungal and antibacterial property (Raghunath and Perumal, 2017). The antimicrobial action of ZnO is related to either disruption of the cell membrane, production of strong oxidizing agents such as hydrogen peroxide (H_2_O_2_) which are lethal and can penetrate bacterial cells, or the generation of reactive oxygen species (ROS) (Xie et al., 2011). On the other hand, the high photocatalytic performance and high photosensitive activity of ZnO nanoparticles owing to its large bandgap, make them an excellent choice to be used for photocatalysis, which has lately emerged as a clean and economical alternate for the industrial treatment of polluted water that contains dyes, i.e., MO (causes environmental pollution and eutrophication of aquatic life) and different types of organic matter (Balcha et al., 2016; Vinayagam et al., 2020).

The biosynthesis approach of the ZnO nanoparticle involves the use of plants or microorganisms as the source material. The synthesis of nanoparticles relies on the capacity of microbes to tolerate the toxicity levels of heavy metals and these microbes synthesize nanoparticles under stress by reducing metal ions to its metal oxide (Mohd Yusof et al., 2019). Thus, zinc-tolerant microorganisms manufacture ZnO nanoparticles by imitating the natural biomineralization method (Jain A. K. et al., 2013). Microorganisms aid in the reduction process of metal ions through the contribution of various biomolecule complexes that are either secreted or produced by the microbes. Considering this perspective, we attempted to synthesize ZnO nanoparticles from the zinc-tolerant bacterial strain *Serratia nematodiphila* and examine their antimicrobial and antifungal potential against phytopathogens. To the best of our knowledge, this is the first report to successfully synthesize ZnO nanoparticles using *Serratia nematodiphila*. Besides, we have evaluated the photocatalytic activity of nanoparticles using an aqueous solution of MO to test its ability to treat polluted water.

## Materials and Methods

The chemicals used in this research were of analytical grade. Chemicals, media, and antibiotic discs were purchased from Sigma Aldrich, SRL, and Hi-Media, respectively. The zinc-tolerant bacterial strain *Serratia nematodiphila* strain ZTB15 (NCBI Genebank accession number MK773869), plant pathogenic bacterial strain *Xanthomonas oryzae* (XO), and plant pathogenic fungal strain *Alternaria alternata* are used in this study.

### Synthesis of ZnO Nanoparticles

*Serratia nematodiphila* strain ZTB15 was inoculated in a Luria Bertani (LB) broth and incubated for 24 h after which it was diluted 4 times with fresh LB broth to the final volume of 100 mL, and again it was incubated for 24 h at 37°C. Further, in this overnight grown culture (OD>1 at 600 nm), 0.1 M zinc sulfate was added through drops and then heated at 80°C for 10 min until a white precipitate appeared at the bottom of the flask and again incubated for 24 h. ZnO nanoparticles were purified by washing through multiple centrifugations at 14,000 rpm for 10 min and dried at 120°C. The dried pellet was preserved for all further characterization and antimicrobial studies.

### Characterization of ZnO Nanoparticles

#### UV-Vis Spectroscopy

ZnO nanoparticles were initially characterized by UV-Vis absorption spectroscopy using a nanophotometer (Implen, Germany).

#### XRD Analysis

The structure and composition of ZnO nanoparticles were characterized by x-ray diffraction (XRD). An X'Pert Pro X-ray diffractometer (PAN analytical BV, The Netherlands) set with voltage maintained at 40 kV and current kept at 30 mA with Cu Kα radiation in θ- 2θ configurations was used. Based on the width of the XRD peaks, the Scherrer formula was used to determine the crystallite domain size with the assumption that they are free from non-uniform strains, the formula:

$$\begin{array}{l} D=\frac{0.94\;\lambda }{\beta \text{ Cos}\theta } \end{array}$$

where D is the average crystallite domain size perpendicular to the reflecting planes, λ is the x-ray wavelength, β is the full width at half maximum (FWHM), and θ is the diffraction angle. To compensate for additional instrumental broadening, the FWHM was corrected, using the FWHM from a large grained Si sample (Sukhwal et al., 2017).

$$\text{β corrected}={\left({\text{FWHM}}_{\text{sample}}^{\text{2}}{\text{-FWHM}}_{\text{si}}^{\text{2}}\right)}^{\text{1/2}}$$

#### Transmission Electron Spectroscopy

ZnO nanoparticles dispersed in water were used for transmission electron microscopy (Tecnai G220 (FEI) S-Twin 200kv) (TEM) analysis. Around 10 μl of sample solution was added onto a carbon-coated copper TEM grid. The grid was dried overnight before TEM analysis. The crystalline nature of ZnO nanoparticles was determined through the selected area electron diffraction (SAED) pattern. The dried ZnO nanoparticles were added onto a double-sided tape and placed directly into the SEM machine (Leica Stereoscan-440 SEM instrument equipped with a Phoenix EDAX attachment) for EDAX measurements based on the images of the nanoparticles obtained.

#### Dynamic Light Scattering (DLS) Analysis and Zeta Potential Measurement

The zeta potential and zeta size distribution of ZnO nanoparticles were determined by the Malvern zeta-sizer nanoseries (UK) to define the size and surface charge of the nanoparticles.

#### Raman Spectroscopy

Raman spectra were acquired on a Horiba LabRAM HR Evolution Raman spectrometer using a 532 nm laser and power of 10 mW on Si/SiO_2_ (300 nm of thermally grown oxide) as substrate.

### Antimicrobial Studies

The antibacterial activities of ZnO nanoparticles were investigated by the disc diffusion method. Luria Bertani plates were prepared, sterilized, and after solidification *Xanthomonas oryzae* culture was swabbed onto these plates. Antibiotic discs were used as positive controls. The sterile disc was dipped in ZnO nanoparticles (25, 50, 75, and 100 μg ml^−1^) and placed in a plate and kept for incubation at 28°C for 24 h. The zone of inhibition was measured and compared with a standard antibiotic disc.

### Antifungal Studies

The different concentrations of ZnO nanoparticles were prepared (50, 100, 150, 200, and 250 μg ml^−1^) from dried powder to measure the antifungal activity using the poison food technique (Saharan et al., 2013). Briefly, a potato dextrose agar medium was prepared with the above mentioned concentrations of ZnO nanoparticles separately and the uniform mycelial bits (diameter, 4.0 mm) taken from the periphery of the *Alternaria sp*. were kept in the center of the petri plate and left for incubation for 5 days. The formula used to determine the pathogen's percent inhibition level is

$$\text{Inhibition rate }\%=\frac{\text{Mc}-\text{Mt}}{\text{Mc}}\text{x}100$$

where, M_c_ = mycelial growth (control) and M_t_ = mycelial growth (treatment).

Further, the antifungal effect of nanoparticles at different concentrations (50, 100, 150, 200, and 250 μg ml^−1^) was also studied on the spore germination of *Alternaria* sp. on glass slides under sterile conditions. Spore suspensions of *Alternaria* sp. were prepared aseptically with a pure culture that was 7 days old. A drop (40 μl) of spore suspension and a drop (40 μl) of different concentrations of ZnO nanoparticles were used and compared with control devoid of any nanoparticle's solution. All treatments were maintained at an ambient temperature (28 ± 2°C) for 8–12 h. The experiment was performed in triplicates, and the data were analyzed under a light microscope. The percentage of the inhibition rate was calculated by totaling the spores germinated when related to control using the formula below.

$$\text{Inhibition rate }\%=\frac{\text{Tc}-\text{Tt}}{\text{Tc}}\text{x 100}$$

where, T_c_= germination in control and T_t_ = germination in treatment.

#### Statistical Analysis

Standard deviation for the experiments were analyzed on Microsoft Excel. The significant difference was analyzed using the JMP software version 11 using the Turkey–Kramer HSD test at *p* = 0.05.

### Photocatalytic Activity of ZnO Nanoparticles

To probe the photocatalytic degradation performance of ZnO nanoparticles, an aqueous solution of an azo dye, MO, was used and the catalytic activity was investigated under the influence of UV light irradiation. The set-up involved adding 100 mg of ZnO nanoparticles in 100 mL of MO aqueous solution (5 mg L^−1^, pH 8.06) and stirring under the influence of a magnetic stirrer for 40 min which would enable the solution to reach equilibrium between the dye molecules and the surface of the nanoparticles. Besides, the experiment was subjected to UV light irradiation and samples were collected at different periods and analyzed. The change in dye concentration was analyzed through spectroscopy at 450 nm and the percentage of degradation was calculated as per the equation reported earlier (Srikanth et al., 2018).

## Result and Discussion

**Figure 1:**
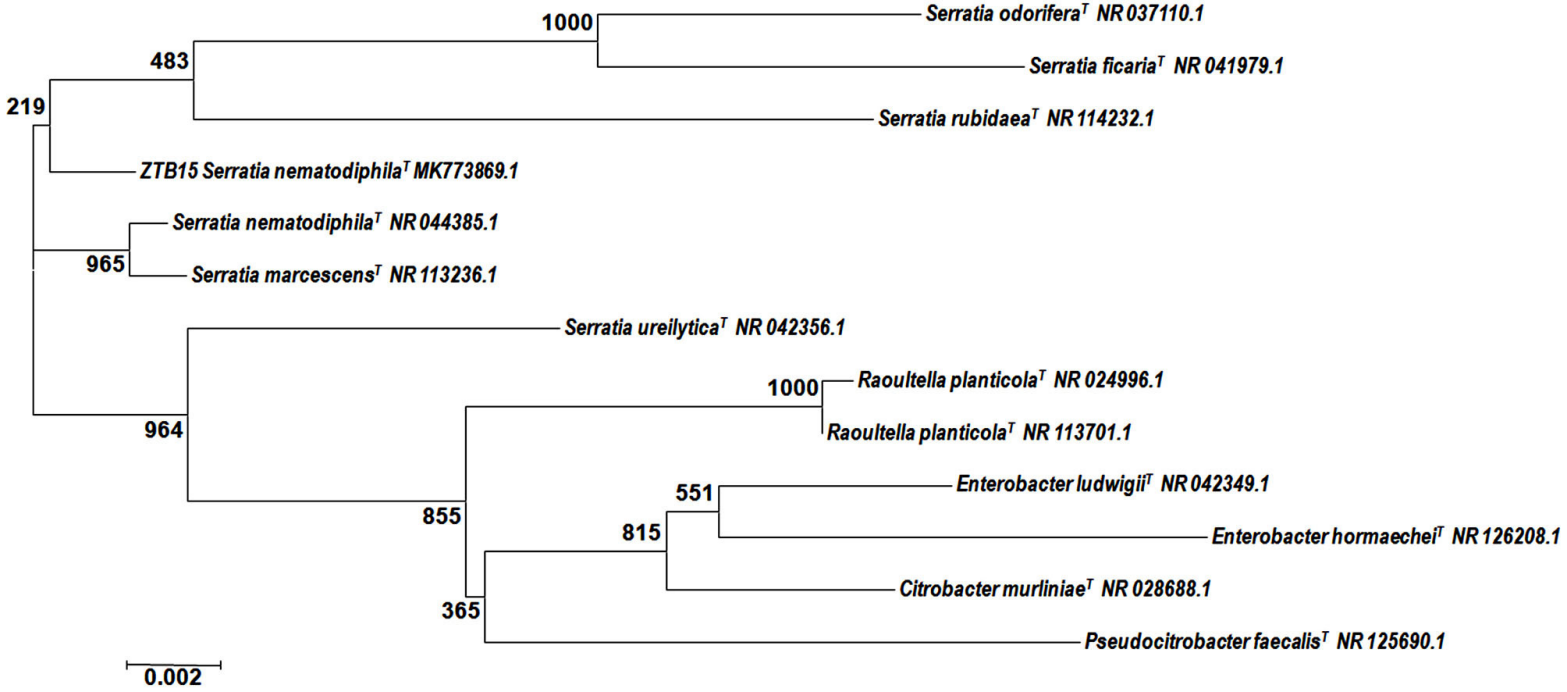
Phylogenetic analysis of the 16S rDNA sequence of the ZTB15 strain used for the green synthesis of zinc oxide nanoparticles.

The zinc-tolerant bacteria (ZTB15) from our previous studies (Kour et al., 2019), which have the capacity to tolerate 63 mM Zn^2+^ ions, were used for the synthesis of ZnO Nanoparticles. Based on the partial 16S rRNA sequencing, ZTB15 was identified as *Serratia nematodiphila* based in their homology and matches with earlier reported bacterial rDNA sequences (Figure 1) and the sequence was submitted to the NCBI Genebank database (Accession number-MK773869). Many microorganisms are known to produce nano-sized minerals and other metallic nanoparticles. These nanoparticles have similar properties to chemically synthesized materials since the microbes regulate the size, shape, and composition of the nanoparticles. The physical properties of the nanoparticles such as the size, shape, and crystallinity are highly regulated by the microbes used in the synthesis process (Krol et al., 2017). Microorganisms such as *Sargassum muticum, Serrbiosatia ureilytica, Candida albicans, Aeromonas hydrophila, Rhodococcus pyridinivorans, Aspergillus fumigates*, and *Alternaria alternata* have all been known to mediate the synthesis process of ZnO nanoparticles (Ahmed et al., 2017). The synthesis of metal and metal oxide nanoparticles relies on the capacity of microbes to endure the toxicity of heavy metals and these microbes synthesize the nanoparticles under stressful circumstances by decreasing metal ions to various metals or nanoparticles of metal oxide (Mohd Yusof et al., 2019). Thus, zinc-tolerant microorganisms manufacture ZnO nanoparticles by imitating the natural biomineralization method shown by the elevated zinc metal tolerance fungus *Aspergillus aeneus* isolated from the zinc mine in India for the extracellular synthesis of ZnO nanoparticles (Jain A. K. et al., 2013).

### ZnO Nanoparticle Synthesis

The successful production of bacteria-modified ZnO nanoparticles can easily be monitored by the presence of a white precipitate, which was an indication of the reaction mixture of the bacteria and the precursor salt, *viz.*, zinc sulfate. As the 0.1 M solution of zinc sulfate was mixed drop by drop to the bacterial culture, the white precipitate was formed and the reaction mixture color changed from colorless to whitish as a consequence of the reduction of zinc ions; which indicated the materialization of ZnO nanoparticles (Supplementary Data Sheet Figure S1). After 24 h the whitish precipitate was obtained which indicated the reduction of zinc ions and the development of ZnO nanoparticles. Studies have shown multiple benefits of the biological synthesis of ZnO nanoparticles. For instance, ZnO nanoparticles synthesized using *Pseudomonas aeruginosa rhamno* lipids (RLs) as the efficient stabilizing agents, with considerably greater antioxidant and free radical scavenging activities makes them promising antioxidants in the biological system (Singh et al., 2014). Similarly, Davaeifar et al. documented that the green synthesis of ZnO nanoparticles using a high purity phycocyanin pigment, which is a bioactive compound of cyanobacteria, could be employed in multiple industrial applications (Davaeifar et al., 2019). Besides, the toxicity of phycocyanin, ZnO nanoparticles are smaller owing to a protective impact on phycocyanin that has decreased the amount of ROS material making them appropriate for therapeutic use.

### Physicochemical Characterization of ZnO Nanoparticles

#### UV-Visible Spectroscopy

**Figure 2:**
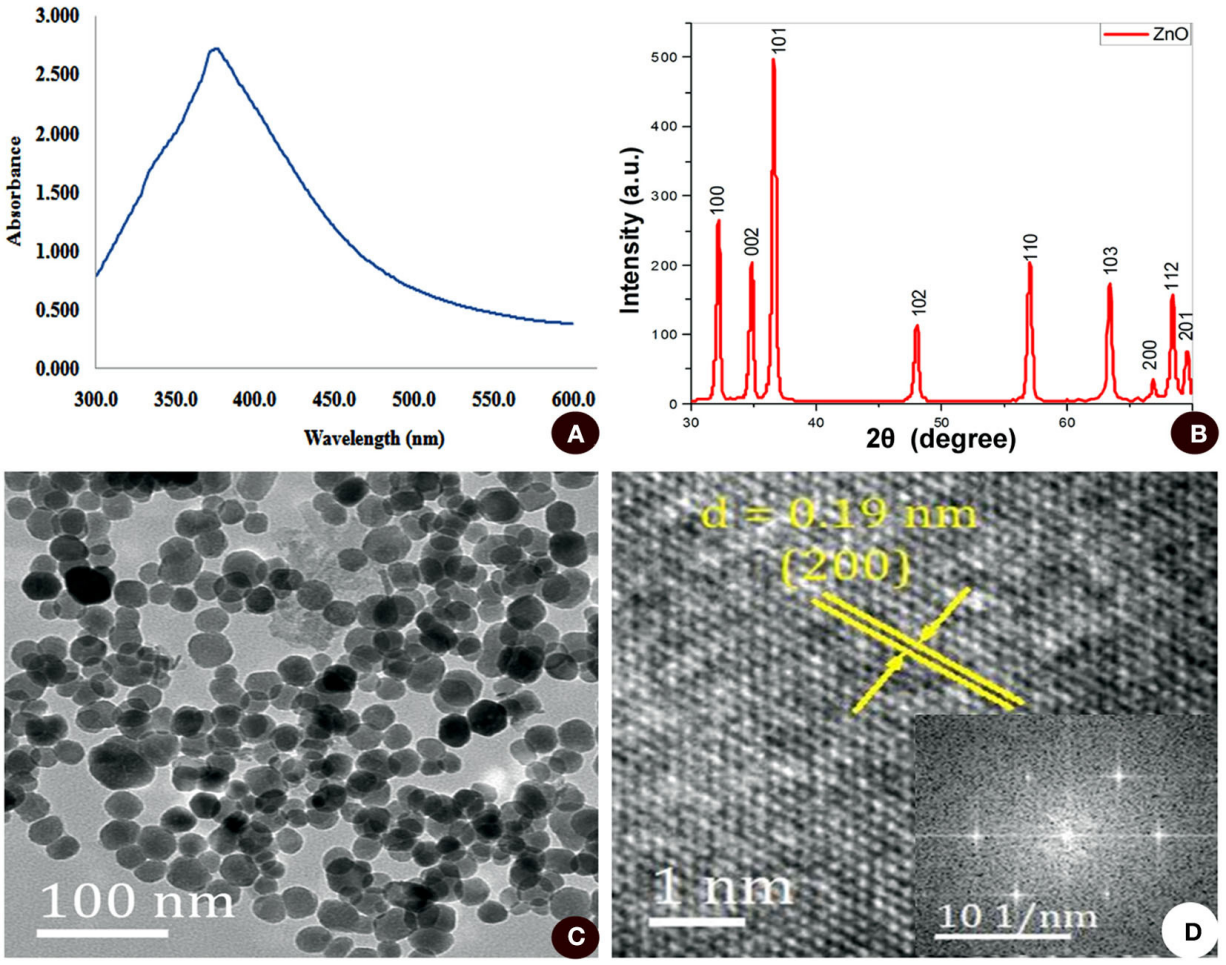
**(A)** UV-VIS absorption spectra from 300 to 600 nm. **(B)** XRD analysis. **(C)** TEM micrograph. **(D)** SAED pattern of ZnO nanoparticles synthesized by zinc-tolerant bacteria.

Here, a preliminary evaluation of synthesized nanoparticles was carried out by UV-visible spectroscopy. The white precipitate obtained from the reaction mixture of zinc-tolerant bacteria and zinc sulfate was dissolved in Milli-Q water and the spectroscopy was read. The highest absorbance of 379 nm was recorded which confirms the transformation of the initial material (zinc sulfate) to the final product (ZnO nanoparticles), as illustrated in Figure 2A. The broadening of the peak also indicated that the ZnO nanoparticles had a polydispersity property of the distribution of nanoparticles (Zhang et al., 2008). These data were in line with multiple studies (Jain A. K. et al., 2013; Selvarajan and Mohanasrinivasan, 2013; Bajpai et al., 2016). The impact of surface plasmon resonance (Sharma et al., 2010) was the result of oscillation of electrons in the conduction band at a specific wavelength. Metal and metal oxide nanoparticles have a UV-visible area intake of SPR between 200 and 600 nm (Sharma et al., 2010). From the UV spectra obtained in the present study, SPR absorption for the sample obtained at 379 nm confirmed the synthesis of ZnO nanoparticles. The chemicals and enzymes present in the zinc-tolerant bacteria reduce Zn^2+^ ions to Zn atoms at first, after which nucleation is encouraged and reduces the remaining Zn^2+^ to their oxides (ZnO) and their additional development leads to the formation of clusters (Chauhan et al., 2015).

#### XRD Analysis

The structure and composition of biosynthesized ZnO nanoparticles were further confirmed by XRD analysis. A typical XRD pattern (Figure 2B) with intense peaks revealed that the ZnO nanoparticles were of high purity and crystalline. The peaks at 2θ= 31.7721, 34.4201, 36.2561, 47.5411, 56.6021, 62.8581, 66.3841, 67.9531, 69.0941, 72.5631, and 76.9671 were assigned to (100), (002), (101), (102), (110), (103), (200), (112). Through comparison with standard data, JCPDS (file no. 043-0002), the synthesized nanoparticles were of hexagonal phase (lattice structure). XRD results clearly showed that the ZnO nanoparticles formed by the bacteria are crystalline, observed by the occurrence of structural peaks in the data, bearing a crystalline size of 24.79 nm corresponding to (101) a peak with a size range of 10 to 50 nm derived from the FWHM of peak observed in the XRD spectrum. The average particle size of the nanostructures was also matched well with the TEM results. The XRD spectrum line extension of diffraction peaks is an indication of the nano-meter range of the synthesized products, moreover, the diffraction peaks from other metal species was absent suggesting the synthesized ZnO nanoparticles are free from any other impurities. Similar results of the XRD analysis of ZnO nanoparticles synthesized by using biological material are in close agreement with multiple studies (Azizi et al., 2014; Nazir et al., 2018; Sharmila et al., 2018).

#### Imaging

Furthermore, TEM analysis was carried out to determine the morphology of the synthesized ZnO nanoparticles. As illustrated in Figure 2C, ZnO nanoparticles were polydispersed and roughly spherical in shape. The size of the nanoparticles ranges from 15 to 30 nm with a mean diameter of 23.09 ± 4.23 nm. The crystalline nature of ZnO nanoparticles is reflected in SAED spots corresponding to the hexagonal crystalline structure and the nanoparticles obtained were highly crystalline in structure, depicted through specific bright spots as observed in the SAED pattern conforming to the preferred nanocrystal orientation (Figure 2D).

The large hydrodynamic size could be due to the inter-particle interactions, viz., van der Waals, and electronic forces which may cause ZnO nanoparticles in an aqueous medium to aggregate. In the present study, the size of the ZnO nanoparticles is very similar to the biosynthesized ZnO nanoparticles by the green synthesis route summarized in Table 1. As observed from the table, our results show that the synthesized nanoparticles are much smaller in size compared to other studies. A smaller size would mean greater surface reactivity and as a result more enhanced properties that could be exploited for multiple uses. SEM analysis showed similar results compared to the TEM data (Supplementary Data Sheet). An energy dispersive x-ray (EDX) of ZnO nanoparticles was carried out by dropping ZnO nanoparticles onto Si (111) wafers and observing them. Zinc and other elements were present in the bacterial cells or secretions and importantly no other elemental impurities were found in the synthesized ZnO nanoparticles (Supplementary Data Sheet). EDX also revealed that ZnO nanoparticles contain organic elements that might facilitate both as a reducing and as a stabilizing agent during the synthesis of ZnO nanoparticles (Supplementary Data Sheet).

**Table 1:** Green synthesis of ZnO nanoparticles using various microbes.

**Microbe**	**Name of the microorganism**	**Particle size (nm) and morphology**	**References**
Bacteria	*Pseudomonas putida*	44.5 nm and spherical morphology	Jayabalan et al., 2019
	*Pseudomonas aeruginosa*	35 to 80 and spherical	Singh et al., 2014
Fungi	*Fusarium keratoplasticum*	10 to 42 nm and hexagonal	Mohamed et al., 2019
	*Aspergillus niger*	8–38 nm and nanorod	Mohamed et al., 2019
Algae	*Spirulina platensis*	40-50 and spherical	Ali et al., 2015
	*Sargassum muticum*	30-57 and hexagonal	Azizi et al., 2014

#### DLS Analysis

**Figure 3:**
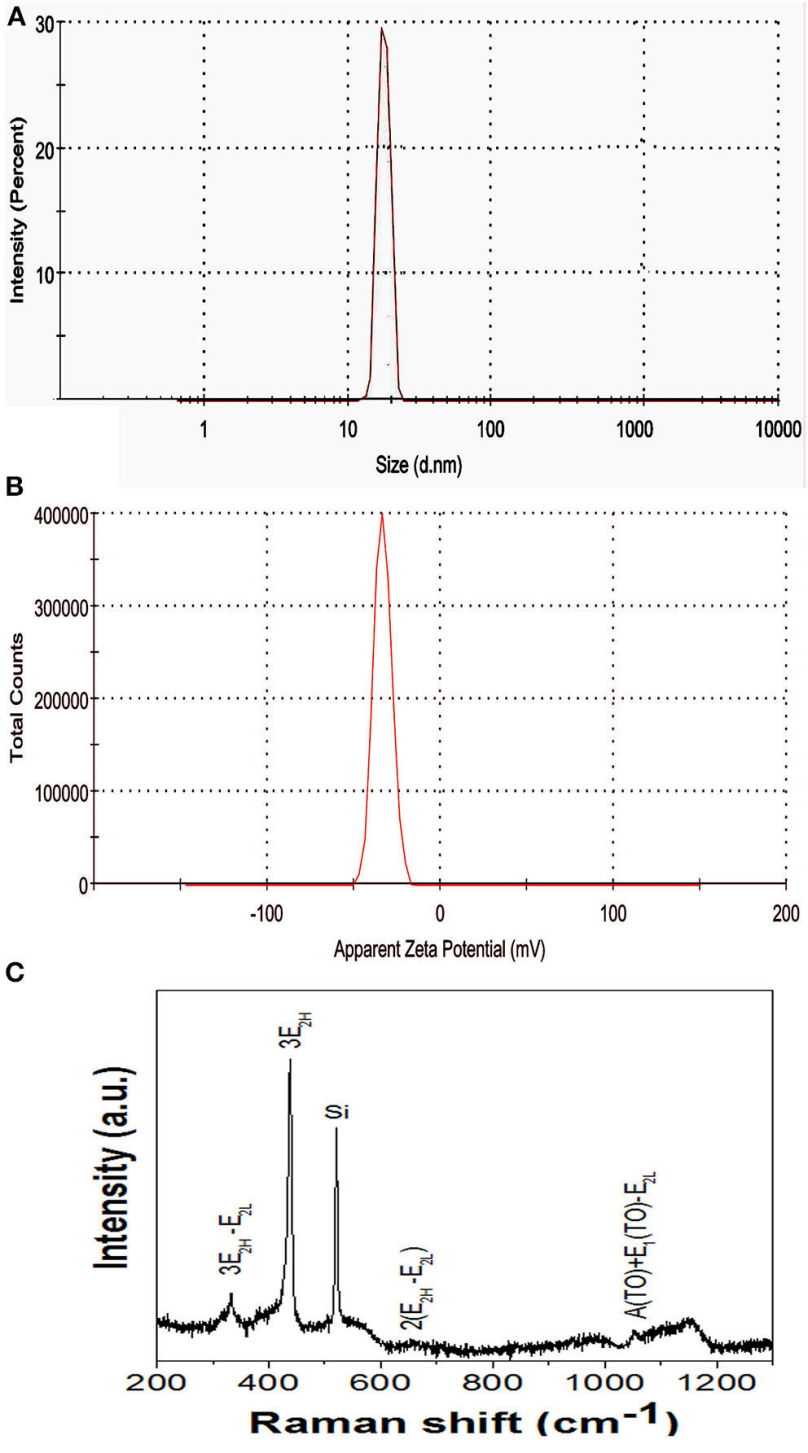
**(A)** DLS analysis and **(B)** Zeta potential analysis. **(C)** Raman spectra of ZnO nanoparticle synthesized using ZTB.

Both the hydrodynamic size and stability of the nanoparticles suspended in solution were evaluated. Nanoparticles were uniform in size ranging from 10 to 30 nm. DLS represents an average particle size and particle size distribution as well as a polydispersity index of the synthesized ZnO nanoparticles. The solution contained particles of uniform sizes ranging from 10 to 30 nm and the average particle size was ~18 nm (Figure 3A). The DLS results were well-supported by TEM studies. The other broad size distribution peak observed at 900 nm could be due to the aggregation of nanoparticles or may be due to the presence of bacterial cell artifacts. The presence of capping agents such as those of proteins and enzymes which are present in biological materials could also affect the size of the nanoparticles (Fouda et al., 2018). In another study, plant extracts facilitated the synthesis of ZnO nanoparticles showed a size distribution of ZnO nanoparticles of 10 to 120 nm with a maximum size distribution of ~90 nm, illustrating the uniformity of ZnO nanoparticles produced (Sundrarajan et al., 2015). The stability of colloidal solutions were determined by a zeta potential assessment based on the surface charge of the produced nanoparticles. The zeta potential correlates to the stability of the nanoparticles in solution. In our study, a zeta potential value of −33.4 mV indicated that the nanoparticles synthesized were highly stable (Figure 3B). DLS measurement of *Pseudomonas aeruginosa-*synthesized ZnO nanoparticles with a mean hydrodynamic diameter was 81 nm, potentially owing to either the agglomeration of ZnO nanoparticles or the hydrodynamic radii of ZnO nanoparticles including the solvent layer for stability (Singh et al., 2014).

#### Raman Spectroscopy Analysis

The purity, crystallinity, structural disorders, and defects in the synthesized ZnO nanoparticles were studied using Raman spectroscopy (Figure 3C.). Having a hexagonal wurtzite structure, according to the group theory, the various optical modes are given as per Equation 1 (Sharma et al., 2012):

(1) $${\text{A}}_{1}+2{\text{B}}_{1}+{\text{E}}_{1}+2{\text{E}}_{2}$$

Here B_1_ modes are silent, A_1_ and E_1_ are polar modes, both Raman and infrared active, E_2_ modes are non-polar, and Raman active only. We observe the basic photon modes of hexagonal ZnO structure at 438.5 cm^−1^ which represents the E_2H_ mode assigned to oxygen vibrations and is the characteristic band of the wurtzite ZnO structure. The multi-photon scattering modes are presented at 332 cm^−1^, 658 cm^−1^, and 1,054 cm^−1^ corresponds to 3E_2H_-E_2L_, 2(E_2H_-E_2L_), and A(TO)+E_1_(TO)-E_2L_, respectively. The phonon mode at 1,154 cm^−1^ represents the acoustic combination of A_1_ and E_2_ modes.

### Antimicrobial Potential of ZnO Nanoparticles

#### Antibacterial Activity

**Figure 4:**
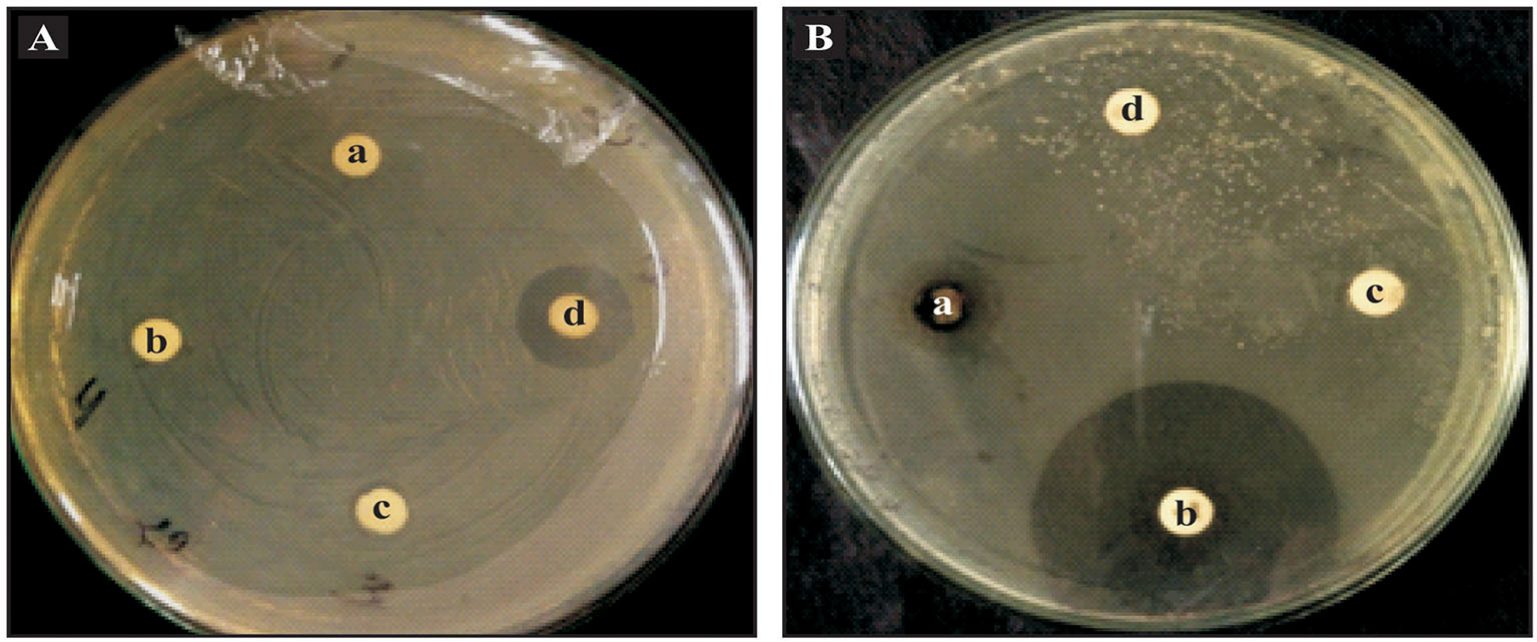
Antibacterial activities of ZnO nanoparticles against *Xanthomonas oryzae*. **(A)** Disc diffusion method with ZnO nanoparticles concentration of (a) 25 μg ml^−1^; (b) 50 μg ml^−1^; (c) 75 μg ml^−1^; and (d) 100 μg ml^−1^. **(B)** Antibacterial activity of ZnO nanoparticles along with standard antibiotics (a) ZnO nanoparticles 100 μg ml^−1^; (b) Rifampicin 5 μg ml^−1^; (c) Nystatin 50 μg ml^−1^; and (d) Penicillin 10 μg ml^−1^.

Post characterization antibacterial activities of ZnO nanoparticles were tested by both the disc diffusion method and poison food technique using a Luria Bertani agar against the bacteria phytopathogen, *Xanthomonas oryzae pv. oryzae* (Figure 4A). The sterile disc was dipped in ZnO nanoparticles (25, 50, 75, and 100 μg ml^−1^) and placed on an LB agar plate and swabbed with the *Xanthomonas oryzae*. A ZnO nanoparticles disc contacting 100 μg ml^−1^ concentration exhibited antibacterial activity against *Xanthomonas oryzae pv. oryzae*. It displayed a clear inhibition zone, however at lower concentrations they were not able to produce clear inhibition zones. In another experiment the ZnO nanoparticles antibacterial activity was compared with standard antibiotic discs (Figure 4B). The ZnO nanoparticles (100 μg ml^−1^ concentration) exhibited antibacterial activity against *Xanthomonas oryzae pv. oryzae* and exhibited a clear inhibition zone. *Xanthomonas oryzae pv. oryzae* was resistant to penicillin (10 μg ml^−1^) as it does not produce inhibition zones and a Nystatin disc (50 μg ml^−1^) was used as a negative control.

ZnO nanoparticles exhibited antimicrobial activities against several pathogenic bacteria, *viz., Pseudomonas aeruginosa, Proteus mirabilis, Bacillus subtilis, Staphylococcus epidermidis, Klebsiella pneumonia, Streptococcus pyogenes, Enterococcus faecalis, Proteus vulgaris, Salmonella typhimurium, Shigella flexneri, Pseudomonas alcaligenes*, and *Enterobacter aerogenes* (Raghupathi et al., 2011; Ibrahem et al., 2017; Mohammadi and Ghasemi, 2018). Bala *et al*. examined the antimicrobial properties of ZnO nanoparticles synthesized *via* green routes against Gram-negative *Escherichia coli* and Gram-positive *Staphylococcus aureus* and the data showed MIC against *E. coli* and *S. aureus* were at concentration of 50 and 100 μg ml^−1^, respectively, which are in close agreement with the concentration used in the present study (Bala et al., 2015). Bhuyan et al. observed the antibacterial activities of green synthesized ZnO nanoparticles against *S. aureus, Streptococcus pyogenes*, and *E. coli* and reported a decrease in bacterial growth with 100 μg ml^−1^ which was also evident in the present data as well (Bhuyan et al., 2015). ZnO nanoparticles also improved the permeability of the bacterial membrane due to its abrasive texture, which ultimately led to the disturbance and disorganization of the bacterial membrane responsible for the modifications in the proteins (Padmavathy and Vijayaraghavan, 2008).

#### Antifungal Activity

**Figure 5:**
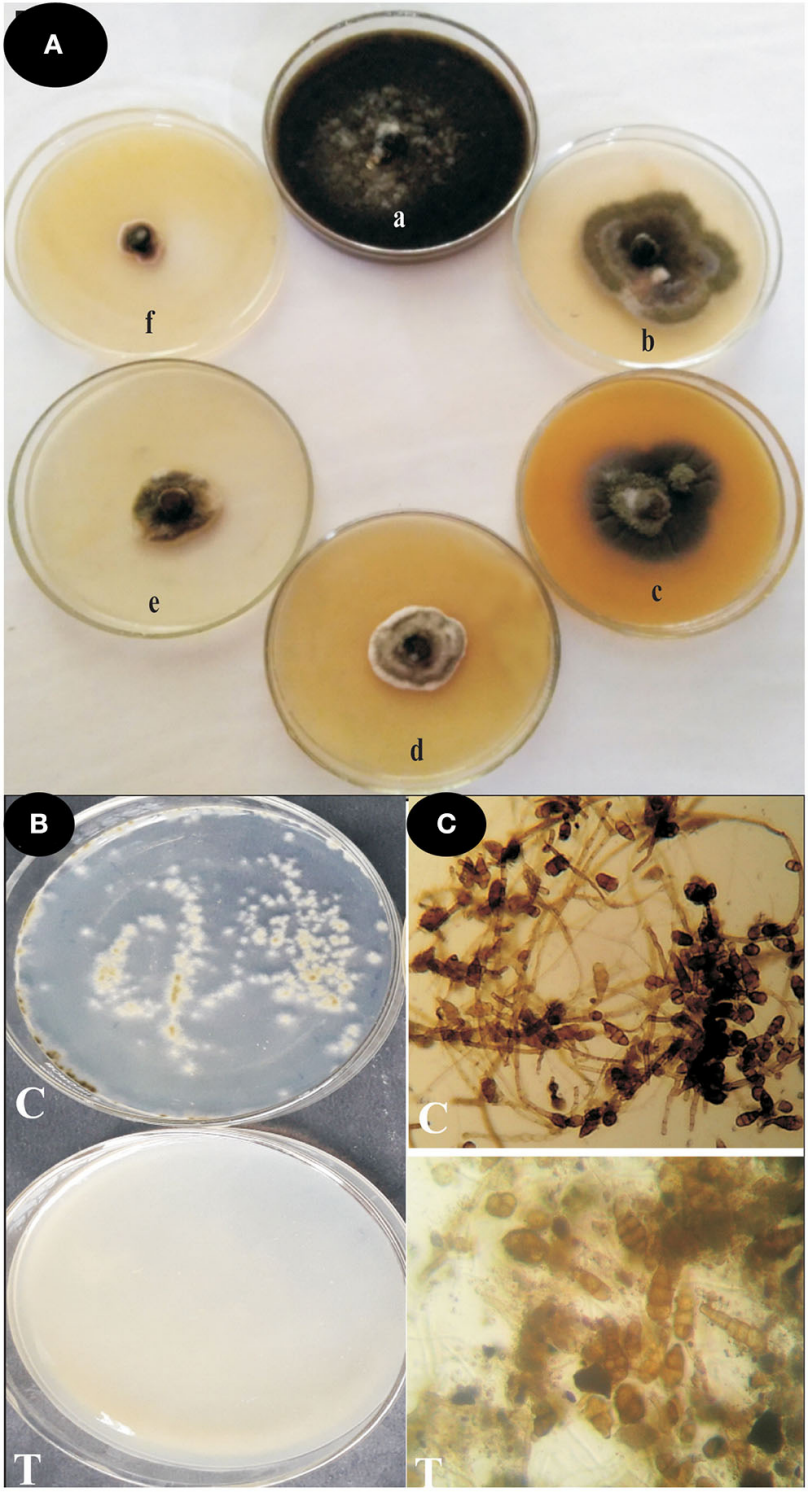
Antifungal activities of green-synthesized ZnO nanoparticles against *Alternaria* sp. **(A)** Effect of ZnO nanoparticles on mycelial growth by poison food technique (a) Control; (b) 50 μg ml^−1^; (c) 100 μg ml^−1^; (d) 150 μg ml^−1^; (e) 200 μg ml^−1^; and (f) 250 μg ml^−1^ZnO nanoparticles. **(B)** Effect of ZnO nanoparticles on spore germination inhibition by pour plate technique C = crude spore suspension & T = 250 μg ml^−1^ZnO nanoparticles. **(C)** Microscopic studies of spore germination inhibition assay C = Control, T = 250 μg ml^−1^ ZnO nanoparticles.

The antifungal activity of ZnO nanoparticles was set up against *Alternaria* sp. by the poison food technique method. The effect of ZnO nanoparticles on spore germination of *Alternaria* sp. was also performed. ZnO nanoparticles demonstrated significant antifungal activity against the phytopathogenic fungi *Alternaria* sp. (Figure 5, Table 2). The radial mycelia growth of *Alternaria* sp. was lowered by different concentrations of ZnO nanoparticles (50, 100, 150, 200, and 250 μg ml^−1^) used in the PDA media as a dose dependent effect. At 85.93% the largest inhibition of mycelia growth was observed at 250 μg ml^−1^ of ZnO nanoparticles. Similarly, *Alternaria* spore germination was also inhibited by the different concentrations of ZnO nanoparticles (50, 100, 150, 200, and 250 μg ml^−1^) as a dose dependent method. The maximum inhibition at 92.22% in the *Alternaria* spore germination was observed at 250 μg ml^−1^ concentration of ZnO nanoparticles. In the pour plate technique, no spore germination and mycelia growth were observed when spores were serially diluted and spread on a PDA medium containing ZnO nanoparticles (250 μg ml^−1^).

**Table 2:** Effect of varying concentrations of ZnO nanoparticles on *in vitro* mycelial growth and spore germination of phytopathogenic fungi *Alternaria*.

**Treatment (ZnO nanoparticles)**	**% Inhibition mycelia growth**	**% Inhibition spore germination**
Control	ND	ND
50 μg ml^−1^	21.67 ± 0.71^*^	18.18 ± 1.45^*^
100 μg ml^−1^	41.44 ± 0.21^*^	36.84 ± 1.51^*^
150 μg ml^−1^	60.07 ± 010^*^	54.54 ± 1.73^*^
200 μg ml^−1^	69.96 ± 0.15^*^	69.47 ± 0.77^*^
250 μg ml^−1^	85.93 ± 0.25^*^	92.22 ± 1.03^*^

* *Each value is a mean of 3 replicates from 2 experiments. Mean ± SD followed by the same letter in a column of each treatment is not a significant difference at p = 0.05 by the Tukey–Kramer HSD test, % inhibition rate was calculated compared to the germination of the control (0%). Control without any formulation. ND, Not defined*.

Jayaseelan et al. studied the antifungal activities of bacteria-assisted ZnO nanoparticles against *A. flavus, A. niger*, and *C. albicans* and concluded that at 25 μg ml^−1^ concentration, ZnO nanoparticles inhibited the mycelia growth (Jayaseelan et al., 2012). Arciniegas-Grijalba et al. described the antifungal activity of ZnO nanoparticles against the coffee fungus *Erythricium salmonicolor* and found that ZnO nanoparticles significantly inhibited the growth of *E. salmonicolor* (Arciniegas-Grijalba et al., 2017). Also, the antifungal effect of ZnO nanoparticles against the pathogenic fungus *Penicillium expansum* was examined and the minimum inhibitory concentration was 9.8 mM (i.e., 798 ppm) which was very high compared to the results observed in the present study (Sardella et al., 2018). Earlier reports suggest that the antifungal activity of ZnO nanoparticles could be facilitated through ROS since the hydroxyl radicals formed in water suspensions of ZnO could be responsible for the cytotoxic effect of ZnO nanoparticles (Lipovsky et al., 2011). Another factor could be the release of Zn^2+^ by ZnO nanoparticles when they come into direct contact with the cell membranes. The negative charge of the cell membrane attracts the positive charge of Zn^2+^ ions which further reacts with the sulfhydryl groups of proteins inside the cell membrane and damages the activity of synthetase. Due to this, the microbes lose their ability of cell division and growth, which causes the death of the microbes (Zhang et al., 2007).

### Photocatalytic Degradation Potential

**Figure 6:**
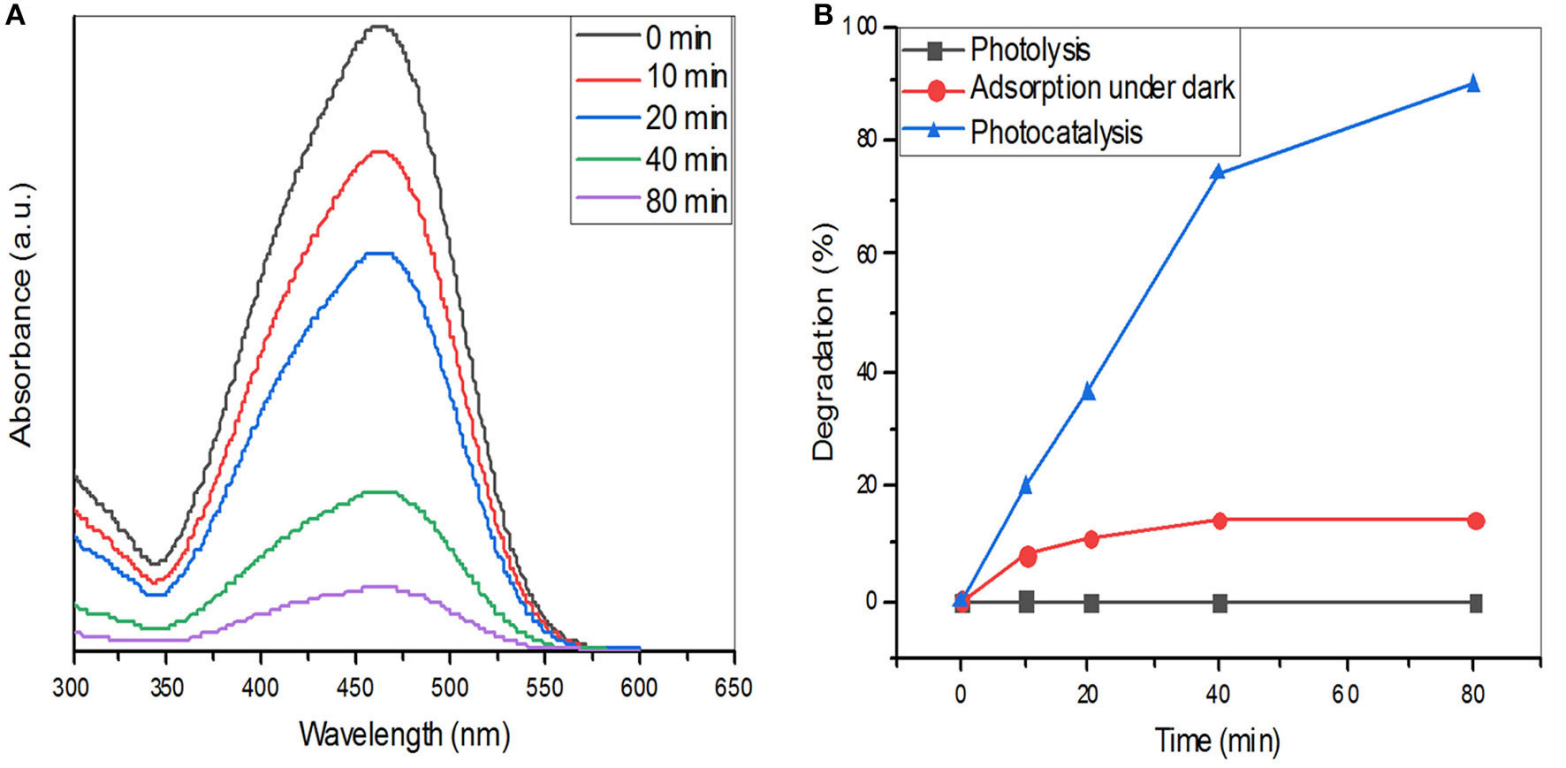
**(A)** UV–vis absorption spectra of reactive methyl orange dye in the presence of ZnO nanoparticles. **(B)** Percentage degradation of methyl orange dye with and without ZnO nanoparticles.

The photocatalytic degradation of MO dye using ZnO nanoparticles as catalysts was examined. ZnO nanoparticles were suspended in MO dye and placed in the dark for 30 min to achieve adsorption-desorption equilibrium and immediately the photocatalytic degradation experiments were carried out under UV light irradiation. Figure 6A shows the variation in absorbance of MO dye against irradiation time with ZnO nanoparticles. It is evident that the peak intensity of MO (463 nm) decreases consecutively with time under the exposure of UV light. A study that synthesized ZnO nanoflowers using *Calliandra haematocephala* (Vinayagam et al., 2020) showed that synthesized nanoparticles had an excellent photodegradation ability against methylene blue dye and they concluded that 88% of the dye could be degraded within 270 min. A similar study using a leaf extract of Cyanometraramiflora to synthesize ZnO nanoparticles showed that the degradation efficiency of pollutant dye Rhodamine B was 98% within 200 min under the influence of sunlight irradiation (Varadavenkatesan et al., 2019). In our study, after 80 mins, ~ 90% of MO degradation was observed which was evident in both the absorbance peak intensity at 463 nm and the visual color observation. According to the Beer-Lambert law, absorbance intensity is proportional to the concentration of moieties in solution and our data show that with increasing time, absorbance of the solution decreases and percentage degradation was increased. When MO dye alone was subjected to photolysis (degradation under UV light), no change in dye concentration was observed. Similarly, when ZnO nanoparticles were subjected in the absence of UV light with the MO solution, only a small change in the concentration of MO was detected as a result of the adsorption of dyes on the surface of nanoparticles. However, when ZnO nanoparticles-treated dye were subjected to photocatalysis under UV light, substantial dye degradation was observed. These data highlight the photocatalytic ability of ZnO nanoparticles for pollutant degradation (Figure 6B).

## Conclusions

In conclusion, we successfully synthesized ZnO nanoparticles using the green route by employing the zinc-tolerant bacteria *Serratia nematodiphila* as the reducing agent. ZnO nanoparticles were well-characterized and a uniformly spherical nanoparticle with a size range of 10–50 nm was produced. The rapid protocol which can be easily reproduced would be advantageous if the technology needs to be transferred to industries. ZnO nanoparticles showed good antimicrobial activity against *Xanthomonas oryzae pv. oryzae* and *Alternaria sp* further enhanced the applicability of the synthesized nanoparticles. Besides, the photocatalytic ability of ZnO nanoparticles could be exploited in environmental protection strategies.

## Data Availability Statement

All datasets generated for this study are included in the article/Supplementary Material.

## Author Contributions

DJ and AS designed the experiment. S, AB, HS, HD, and MS carried out the experiment. BS, DJ, and AS wrote the manuscript with support from SM, HD, and MS. All authors discussed the results and contributed to the final manuscript.

## Conflict of Interest

The authors declare that the research was conducted in the absence of any commercial or financial relationships that could be construed as a potential conflict of interest.
