# Supplementary material for: Microbial Fabrication of Zinc Oxide Nanoparticles and Evaluation of Their Antimicrobial and Photocatalytic Properties
Source: Front Chem. 2020 Sep 30;8:778. doi: 10.3389/fchem.2020.00778 (PMC7554571; doi:10.3389/fchem.2020.00778)
Supplement: Supplementary file 1 [file Data_Sheet_1.PDF]

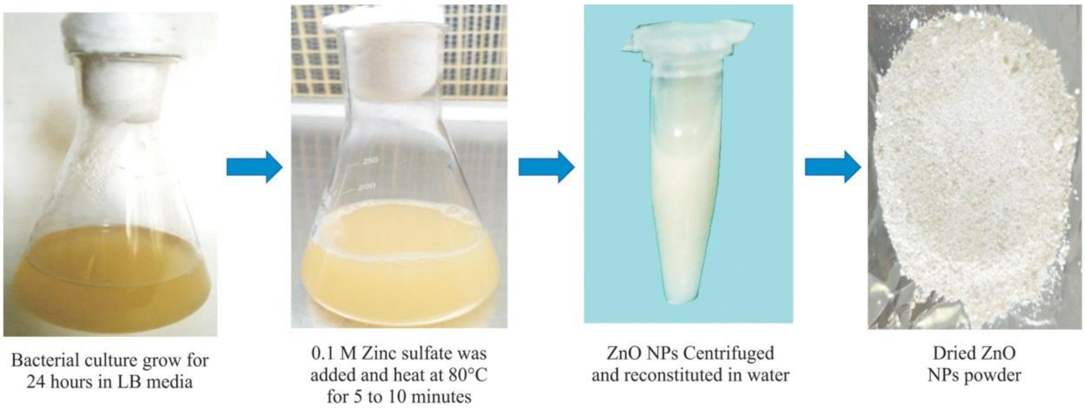

Figure S1: Schematic represention of zinc tolareant bacteria assisted synthesis of ZnO nanoparticles

Figure S2

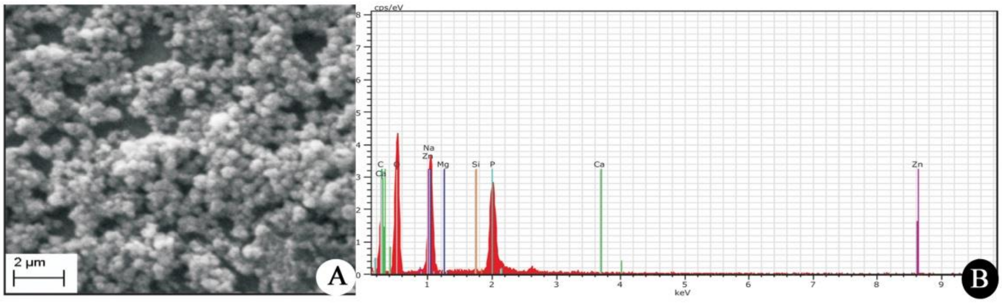

Figure S2: A. SEM micrograph of ZnO nanoparticles B. EDS analysis of ZnO nanoparticles

For SEM assessment the suspended ZnO nanoparticles in sterile Milli Q water were used by manufacturing a drop of suspension on smooth electrical stubs and enabling water to evaporate fully. As shown in Figure S2, the SEM picture of ZnO revealed spherical and comparatively uniform forms of formation of nanoparticles. The few bigger nanoparticles of ZnO shown in SEM picture may be due to lower aggregation. Energy dispersive X-ray (EDX) of the ZnO nanoparticles drop coated onto Si (111) wafers was performed thus exhibiting the presence of the zinc and the other elements which were present in the bacterial cells or secretions and importantly no other elemental impurities present in the synthesized ZnO nanoparticles. The EDX also revealed that the ZnO nanoparticles contain organic elements which might facilitate as both a reducing and as stabilizing agents during the synthesis of ZnO nanoparticles.
